# Starvation Influences the Microbiota in the Stomach of the Corallivorous Crown-of-Thorns Starfish

**DOI:** 10.3390/biology14081102

**Published:** 2025-08-21

**Authors:** Ying Zhang, Fuxiang Lai, Litong Yang, Liling Dai, Nan Su, Jianxing Hu, Huizhen Chen, Qian Gao, Fanyu Zheng, Chang Chen

**Affiliations:** 1 CAS Key Laboratory of Tropical Marine Bio-Resources and Ecology, Guangdong Provincial Key Laboratory of Applied Marine Biology, South China Sea Institute of Oceanology, Chinese Academy of Sciences, Guangzhou 510301, China; zhangy@scsio.ac.cn (Y.Z.); 17779713400@163.com (F.L.); litong.yang@duke.edu (L.Y.); 15280840578@163.com (L.D.); su3310596978@163.com (N.S.); hujx123@126.com (J.H.); chenhuizhen20@mails.ucas.ac.cn (H.C.); gaoqian21@mails.ucas.ac.cn (Q.G.); 17347480708@163.com (F.Z.); 2 Xisha Marine Environment National Observation and Research Station, South China Sea Institute of Oceanology, Chinese Academy of Sciences, Sansha 573199, China; 3 College of Marine Sciences, South China Agricultural University, Guangzhou 510640, China; 4 Institute of Hydrobiology, Jinan University, Guangzhou 510632, China

**Keywords:** coral reefs, outbreaks, 16S rRNA gene, *Endozoicomonas*, *Mycoplasma*

## Abstract

The crown-of-thorns starfish (CoTS, *Acanthaster* spp.), is responsible for a considerable amount of coral loss in the tropical Indo-Pacific region. After decimating coral populations through predation, it is expected that CoTS will face food scarcity before coral recovery. It is unclear how CoTS respond to starvation stress. Therefore, we conducted a four-month starvation stress experiment on CoTS, and analyzed the response characteristics of its stomach bacterial communities using high-throughput 16S rRNA sequencing. The results showed no significant differences in the α-diversity of the stomach bacterial community, but the β-diversity differed significantly between fed and starved CoTS. The bacterial community composition in the CoTS stomach changed dramatically, manifesting mainly from Tenericutes to Proteobacteria at the phylum level, and from a community dominated by digestive bacteria (*Mycoplasma*) to beneficial bacteria (*Endozoicomonas*) at the genus level. Furthermore, co-occurrence network analysis revealed that starvation reduced the network stability of the bacteria community, but improved network modularity, which may have contributed to the CoTS adaptation to chronic starvation. This study provides a basis for elucidating the mechanism of starvation resistance in the crown-of-thorns starfish.

## Simple Summary

Periodic outbreaks of the crown-of-thorns starfish (CoTS), a primary coral predator, have caused severe damage to Indo-Pacific reefs. A critical unknown is how CoTS cope with prolonged starvation at the end of an outbreak to preserve their population for seeding the next outbreaks. Given the role of gastric microbiota in host environmental adaptation, this study investigated the response of CoTS stomach bacterial communities under experimental starvation over four months. The results revealed a taxonomic shift from digestive Mycoplasma (Tenericutes) to beneficial Endozoicomonas (Proteobacteria), accompanied by increased network modularity and reduced stability. These findings highlight the role of stomach bacterial communities in mediating CoTS starvation stress adaptation, laying the foundation for understanding CoTS periodic outbreaks and informing reef restoration strategies.

## 1. Introduction

Coral reefs are the most productive ecosystems in the world, with important ecological, economic and social values [1,2]. Although they occupy less than 0.1% of the ocean, they provide a habitat for about a quarter of all marine life on Earth [3]. However, in recent years, coral reef ecosystems around the world are facing serious threats of degradation and destruction due to increasing mortality rates of hermatypic corals [4]. Among the many factors contributing to coral reef degradation, the outbreak of crown-of-thorns starfish (CoTS; *Acanthaster* spp.) was one of the main causes [5,6]. CoTS belong to the phylum Echinodermata and undergo a dietary shift during their development. For example, juvenile CoTS feed on crustose coralline algae, but after around four-six months they switch to corallivory until they reach sexual maturity [7,8]. This makes the species a primary predator of coral, as it spends most of its time preying on it. It was responsible for 42% of the coral degradation in the Australian Great Barrier Reef over the last 27 years, far more than the 10% bleaching caused by other factors such as warming sea temperatures, disease infections and ocean acidification [4].

Several outbreaks of CoTS have been discovered in the South China Sea in recent years, leading to extensive coral loss ranging from 43% to 97% [5,9]. These outbreaks are not random occurrences, but rather happen periodically every 15–20 years in coral reefs worldwide [10]. After two to five years of intensive coral feeding, an outbreak of CoTS usually ends suddenly, accompanied by a large-scale loss of the CoTS population [5,11]. After 10–15 years of coral recovery, CoTS may return, suggesting that the remaining surviving individuals have started a new cycle of outbreaks [8,12,13].

During periodic outbreaks, large populations of CoTS suddenly disappear en masse after feeding on an area of live coral each time. Moran (1986) suggested that the disappearance of CoTS may be caused by migration in search of alternative habitats triggered by food depletion [14]. Others have attributed it to the mass death of CoTS infected with lethal pathogens under starvation stress [15,16]. However, no mass die-offs of CoTS have been observed in the wild [8,17,18]. In our recent study, it was unexpectedly found that CoTS can survive for up to four months without feeding [19]. We speculated that CoTS may have an anti-hunger mechanism that allows them to maintain their basic metabolism in the absence of food. This mechanism could even help the population survive in the event of food shortage, enabling them to trigger the next outbreak.

Crucially, such extended survival in the face of starvation likely involves symbiotic microorganisms—a strategy that is increasingly recognized as being key to environmental adaption in marine invertebrates. These microorganisms play various roles and functions in the host. They participate in immune regulation, nutrient metabolism, and defense against invading pathogens in the host [20,21]. The gut microbiota plays a diverse unexpected role in maintaining and regulating host animal physiology and enhancing environmental adaptation, particularly in the face of starvation [22]. Bacterial communities may play a role in amino acid uptake on the integument [23], and digestive strategies in the gut of echinoderms [24,25]. Various tissue parts of CoTS are reported to host a variety of bacteria with tissue specificity, which may be crucial for host energy metabolism and survival [26]. For instance, Spirochetes (part of the phylum Spirochaeta) are highly abundant in the body walls and tubular feet of healthy CoTS [27]. This group constitutes the core microbiota of many octocorals, such as *Corallium rubrum* [28] and *Lobophytum pauciflorum* [29], which play a role in host nutrition and the specific microbiota by producing antimicrobial agents. Similarly, studies have reported that phototrophic bacteria located in the gut of CoTS larvae, such as Proteus and Bacteroides, provide the host with phototrophic products and a diverse library of metabolic functions. These bacteria may contribute to the response to oligotrophic status [30]. In addition, research has shown that the helicobacter-related taxon was dominant in the coelomic fluid of two common coastal starfish (*Patiria pectinifera* and *Asterias amurensis*) [31]. This taxon has the ability to oxidize sulfide and may aid in sulfide detoxification [32]. Therefore, we hypothesized that the stomach microbiota of CoTS may also play an important role in the host maintenance of basic energy metabolism in the face of food scarcity. However, it is unclear how the stomach microbiota responds to starvation stress to help the host survive a dangerous period of food deprivation.

In this study, we subjected CoTS to starvation stress for four months and analyzed changes in the stomach bacterial community structure using 16S rRNA high-throughput sequencing technology, and explored the responses of the key bacterial groups. This study is the first to investigate the changes in the stomach bacterial community of CoTS under chronic starvation. The findings will help clarify the mechanisms of population preservation during food scarcity and will lay the foundation for a deeper understanding of the periodic outbreaks of the crown-of-thorns starfish.

## 2. Materials and Methods

### 2.1. Experimental Design and Sampling

**Figure 1:**
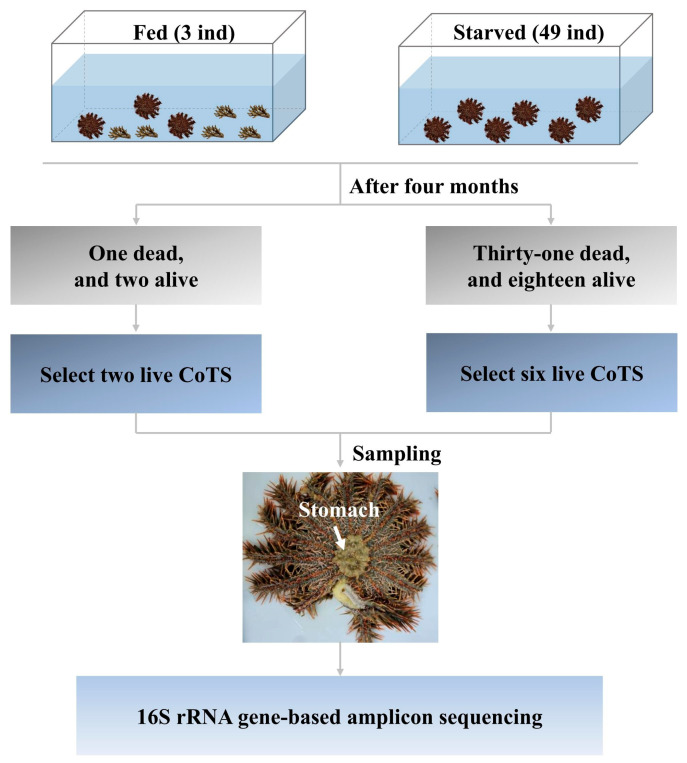
The schematic diagram of the CoTS starvation stress experimental design.

Fifty-two adult CoTS were collected from the South China Sea near Tanmen (18°54′18″ N, 110°51′12″ E) (Hainan, China) in August 2021. They were then transferred immediately to a 4000 L tank with flowing seawater at the Tropical Aquatic Research and Development Center (Hainan, China) to adapt to their new environment for a week, during which they were fed the coral *Acropora Formosa* collected from the wild. After this period, all healthy CoTS were divided into fed and starved groups with a temperature of 23.5 ± 0.8 °C and salinity of 30.4 ± 2.9 ppt. Due to the preciousness of corals and the substantial amount of food consumed by the CoTS, which is estimated to be as much as 12 m^2^ yr^−1^ of coral tissue per individual [33], the fed group consisted of three randomly selected CoTS that were mainly fed with the coral *Acropora formosa*, when the coral was consumed by 80%, new coral was replenished to ensure an adequate food supply for the fed group, while the remaining 49 were starved (starved group). After four months, two CoTS survived in the fed group, while 18 survived in the starved group. Six CoTS that survived starvation were randomly selected from the starved group and two CoTS from the fed group were selected for stomach tissue sampling (Figure 1). The samples were rinsed three times with sterile filtered seawater and then frozen overnight in liquid nitrogen before being stored at −80 °C.

### 2.2. DNA Extraction and Sequencing

The samples were thawed on ice and homogenized using the Motor-Driver Tissue Grinder (Sangon Biotech, ShangHai, China). Genomic DNA was extracted from the homogenized sample using the HiPure Stool DNA Kits (Magen Biotech Corporation, Guangzhou, China) according to the manufacturer’s instructions. The integrity and purity of the DNA were assessed by agarose gel electrophoresis and a NanoDrop 2000 spectrophotometer (Thermo Fisher Scientific, Waltham, MA, USA). The V3–V4 region of the 16S rRNA gene was amplified using specific primers 314F (5′-CCTACGGGNGGCWGCAG-3′) and 806R (5′-GGACTACHVGGGTATCTAAT-3′) with barcodes. The PCR conditions consisted of an initial denaturation step at 95 °C for 5 min, followed by 30 cycles of 95 °C for 1 min, 60 °C for 1 min, and 72 °C for 1 min, and a final elongation step at 72 °C for 7 min. The PCR products (~470 bp) were purified using AM Pure XP Beads and quantified using the ABI StepOnePlus Real-Time PCR System (Life Technologies, Foster City, CA, USA). PCR products were pooled in equimolar amounts and sequenced using an Illumina Hiseq2500 sequencer by Gene Denovo Biotechnology Co. (Guangzhou, China). The sequencing was performed with double-end reads of 2 × 250 bp sequencing.

### 2.3. Bioinformatics and Data Analysis

The raw reads underwent filtering, denoising, and merging to produce raw ASVs (amplicon sequence variants) using the DADA2 R package (version 1.14) [34]. The UCHIME algorithm [35] was employed to identify and remove chimera sequences. The random rarefaction was performed on each sample to obtain an equal sequencing depth (i.e., to obtain the same minimum number of sample sequences). The representative ASV sequences were classified into organisms using the RDP Classifier (version 2.2) [36] based on the SILVA database (version 138.1) [37].

The bacterial community composition was visualized using a stacked bar plot in the R project ggplot2 package (version 4.2.1), based on the relative abundance of the number of all reads assigned to ASV belonging to each taxon (phylum or genus). Alpha diversity was estimated using the Richness, Shannon index, Pielou, and Simpson index, and was calculated using the R project vegan package (version 4.2.1). The statistical significance of the differences in alpha diversity indices between the fed and starved groups was analyzed using the Wilcoxon rank-sum test. Beta diversity was assessed using Bray–Curtis dissimilarity. Principal Coordinates Analysis (PCoA) based on this distance matrix was employed to visualize the differences in stomach bacterial communities in CoTS between the fed and starved groups, with plots generated using the R ggplot2 package (version 4.2.1). To explore whether starvation affected the CoTS stomach bacterial community structure, three nonparametric multivariate statistical methods (Multi-response Permutation Procedure [MRPP], Permutational Multivariate Analysis of Variance [PERMANOVA], and Analysis of Similarities [ANOSIM]) were implemented.

Statistical differences in the relative abundance of bacterial communities in phylum and genus were analyzed using STAMP (Statistical Analysis of Metagenomic Profiles) software (version 2.1.3) based on White’s non-parametric *t*-test. A significance level of *p* < 0.05 was considered statistically significant. Differentially abundant taxa (*p* < 0.02) were shown with 95% confidence intervals. To identify the biomarker ASVs, linear discriminant analysis (LDA) effect size (LEfSe) [38] was applied with a Wilcoxon *p*-value< 0.05 and a logarithmic LDA score > 2. Identified biomarkers were further filtered by FDR < 0.05. Spearman’s correlation coefficient was used to calculate the correlation in the co-occurrence network analysis, and FDR was used for multiple comparison correction at a significance level of 0.05.

## 3. Results

### 3.1. 16S rRNA Gene Composition

**Figure 2:**
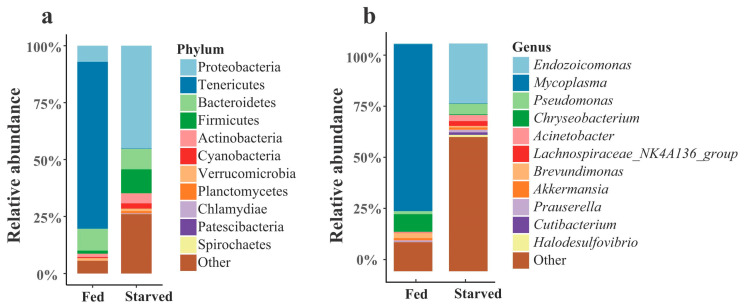
The composition of the bacterial community in the CoTS stomach at the phylum (**a**) and genus (**b**) levels.

A total of 957,163 high-quality clean tags were obtained from eight samples, ranging from 91,074 to 127,373. These were used to identify 2135 amplicon sequence variants (ASVs) using the DADA2 R package (version 1.14). The 16S rRNA gene sequences retrieved from all samples were classified into 25 bacterial phyla. The most abundant phylum was Proteobacteria (35.57%), followed by Tenericutes (18.52%), Bacteroidetes (9.10%) and Firmicutes (8.29%) (Figure 2a and Figure S1a). At the genus level, only five genera were identified with an abundance of 2% or greater, namely *Endozoicomonas* (19.74%), *Mycoplasma* (18.50%), *Pseudomonas* (3.82%), *Chryseobacterium* (2.23%) and *Acinetobacter* (2.06%) (Figure 2b and Figure S1b).

### 3.2. Variation Stomach Bacterial Diversity and Community Structure in CoTS

**Figure 3:**
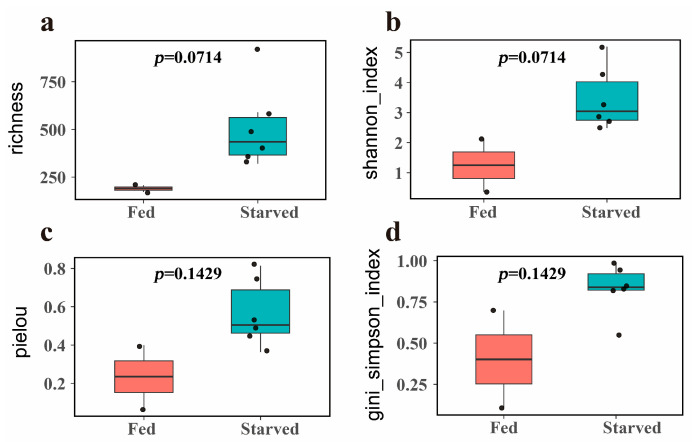
Alpha-diversity boxplot of fed and starved CoTS. (**a**): richness, (**b**): shannon_index, (**c**): pielou, (**d**): gini_simpson_index. The Wilcoxon rank-sum test is used to determine the difference between fed and starved CoTS.

The four alpha diversity indices showed that the richness (Richness), evenness (Pielou) and diversity (Shannon_index and Gini_simpson_index) of bacterial communities in the starved group were higher than those in the fed group (Figure 3; Table 1), but the difference was not statistically significant (*p* > 0.05, Figure 3; Table 1).

**Figure 4:**
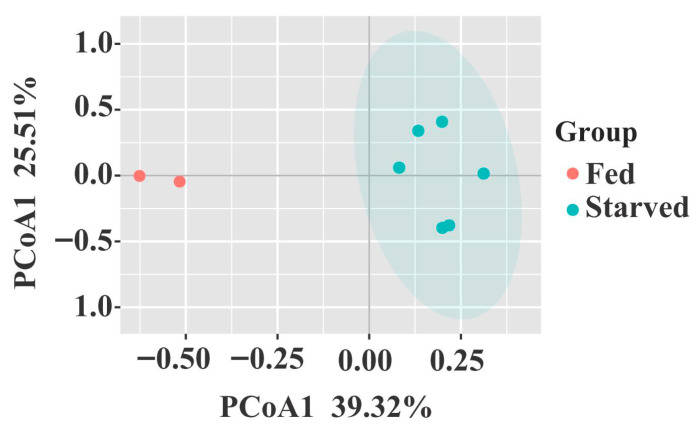
Principal coordinates analysis (PCoA) of the stomach bacterial community structure in fed and starved CoTS.

Principal Coordinates Analysis (PCoA) showed a clear separation between the fed and starved groups in the bacterial community structure (Figure 4). Three nonparametric multivariate statistical methods confirmed a substantial dissimilarity in the overall bacterial community structure between the two groups (*p* < 0.05, Table 2).

### 3.3. Changes in Stomach Bacterial Composition in CoTS Under Starvation Conditions

**Figure 5:**
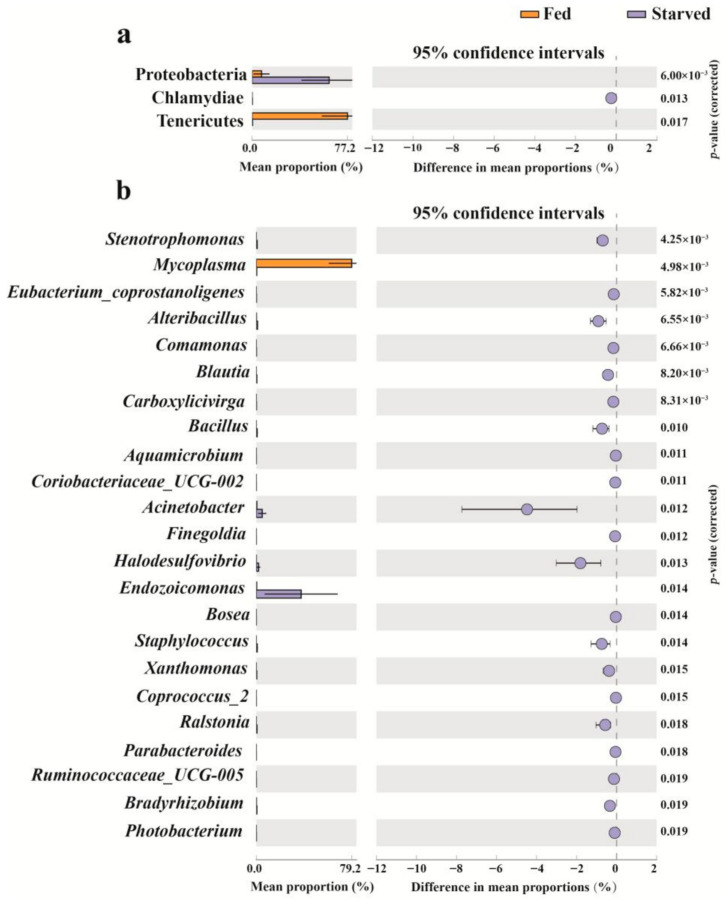
Subsystem enriched or depleted with the bacterial phylum (**a**) and genus (**b**) between fed and starved groups. Differentially abundant taxa (*p* < 0.02) shown with 95% confidence intervals.

STAMP showed a distinct shift in the bacterial community composition of the CoTS stomach during starvation. Specifically, three phyla showed significant changes, namely Proteobacteria, Chlamydiae and Tenericutes (Figure 5a, *p* < 0.05). The Fed CoTS were dominated by Tenericutes (77.20%) with a lesser abundance of Proteobacteria (7.65%) and without Chlamydiae, while a steep decrease in the percentage of Tenericutes (0.33%) and a rapid increase in the percentage of Proteobacteria (62.35%) and Chlamydiae (0.24%) showed in Starved CoTS (Figure 5a, *p* < 0.05).

At the genus level, a total of 22 genera were significantly altered, of which 21 genera significantly increased and only genus *Mycoplasma* decreased in the starved group compared to the fed group (Figure 5b, *p* < 0.05). *Mycoplasma* was identified as the dominant genus in the fed group, accounting for 79.16%, whereas *Endozoicomonas* became the dominant taxon in the starved group, accounting for 37.26%, and the relative abundance of the *Mycoplasma* genera decreased to 0.40%. In addition, there are seven genera with a significant increase in abundance of more than 0.5% in the starved group compared to the fed group, namely *Acinetobacter*, *Halodesulfovibrio*, *Alteribacillus*, *Staphylococcus*, *Bacillus*, *Stenotrophomonas* and *Ralstonia* (Figure 5b, *p* < 0.05).

**Figure 6:**
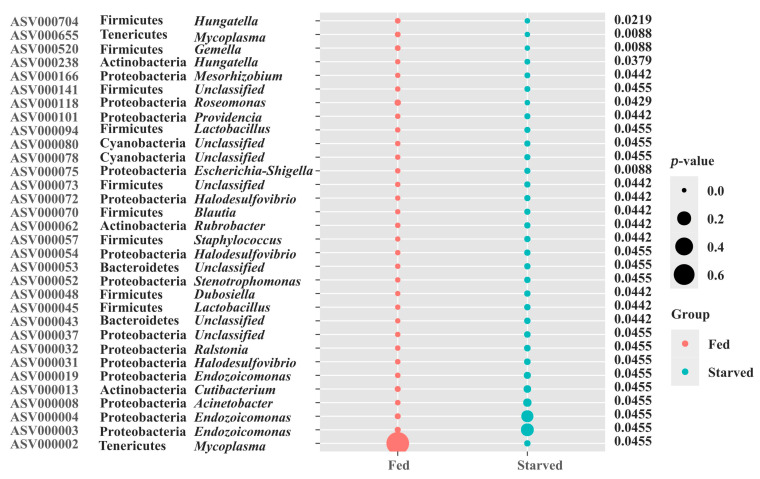
Bubble graph showing relative abundances of biomarker ASVs.

To further clarify which key microbiota members in the CoTS stomach were significantly affected by starvation stress, we applied LEfSe to identify the biomarker ASVs for the Fed and Starved CoTS microbiota. A total of 32 ASVs were identified as potential biomarkers, of which the abundance of 5 biomarker ASVs depleted significantly and 27 ASVs increased significantly after starvation stress (Figure 6). The abundance of ASV000002 and ASV000655, which belong to the genus *Mycoplasma* of the phylum Tenericutes, decreased sharply in the starved group compared to the fed group. Similarly, ASV000520 and ASV000704 which belong to the phylum Firmicutes, and ASV000118 which belongs to the phylum Proteobacteria, also experienced a decrease in abundance (Figure 6). However, three biomarkers, ASV000003, ASV000004 and ASV000019, classified as the genus *Endozoicomonas* of phylum Proteobacteria, significantly increased after starvation treatment (Figure 6). Furthermore, the starved group exhibited a higher abundance of ASV000008 from the genus *Acinetobacter*, ASV000013 from *Cutibacterium*, ASV000031 from the genus *Halodesulfovibrio*, ASV000032 from the genus *Ralstonia*, and 20 other ASVs from 11 different genera compared to the fed group.

### 3.4. Network

**Figure 7:**
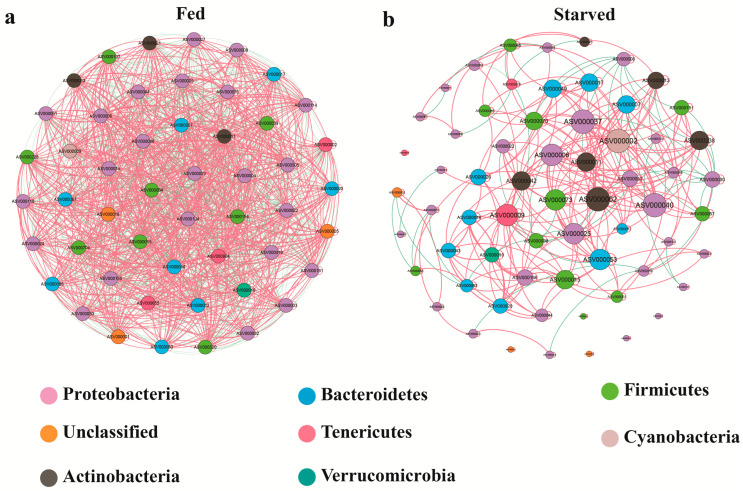
Co-occurrence network analysis of ASVs showing bacterial network patterns in the Fed (**a**) and Starved (**b**) group. Each connection indicates a strong significant correlation, with Spearman’s correlation coefficient ≥ 0.5 and *p* < 0.05. Each node represents an ASV, different phylum with different color, and the size is proportional to the node connectivity. Each edge represents a linkage between two co-occurring nodes, and the color represents a correlation (red is positive, green is negative).

Co-occurrence network analysis was conducted to characterize how starvation affects microbial interactions in the stomach of CoTS. In the fed group, a close interaction relationship was established among the 50 ASVs, resulting in a total of 1275 significant correlations (*p* < 0.05), including 675 positive correlations and 600 negative correlations (Figure 7; Table 3). Following starvation stress, the interactions between microorganisms became noticeably weaker and simpler, with many isolated nodes present. The study found 308 significant correlations among 64 ASVs, with 265 being positive and 43 being negative correlations (Figure 7). Additionally, the average degree of co-occurrence network decreased from 51 to 9.625 after the starvation treatment (Table 3).

Based on nodes with high values of degree (≥10) and closeness centrality (>0.3), the network hubs were further defined in the occurrence network. The number of network hubs was found to be significantly different between the Fed and starved groups. In the fed group, 50 network hubs were found (Supplementary Table S1), while after four months of starvation, the number of network hubs dropped to 31 (Supplementary Table S2). However, the modularity was found to be 0 in the fed group, whereas it was 0.559 in the Starved group (Table 3). This suggests that the co-occurrence network of stomach microorganisms became more modular after experiencing starvation stress.

## 4. Discussion

Symbiotic relationships with bacteria are common among marine vertebrates and invertebrates, including all five echinoderm taxa: Holothuroidea, Ophiuroidea, Crinoidea, Echinoidea and Asteroidea [21,39,40]. The stability of this relationship is crucial for maintaining host health, including nutrition provision, developmental support, immune system promotion and infection prevention [22,41]. However, it is susceptible to environmental factors, with starvation being one of the most common situations it faces [42]. This study is the first to explore the changes in bacterial community composition and structure in the stomach of CoTS after four months of starvation. The results demonstrate that chronic starvation significantly alters the microbial community structure in the CoTS stomach, which may enhance the adaptability of CoTS to starvation and maintain their survival.

### 4.1. Changes in the Bacterial Community Composition and Diversity in the CoTS Stomach

The study analyzed four alpha diversity indices, namely Richness, Pielou, Shannon-index and Simpson-index, in both starved and fed CoTS. The results showed that the starved CoTS had higher values for all four indices, but the difference was not statistically significant, possibly due to the limited sample size [43]. However, a significant difference was found in the β-diversity between the two groups, which is consistent with the results of other studies where starvation has been shown to alter the community structure of intestinal microbiota in grass carp (*Ctenopharyngodon idellus*) [44], largemouth bass (*Micropterus salmoides*) [45], and golden pompano (*Trachinotus ovatus*) [46]. Similarly to previous findings [30], the starved CoTS stomach bacteria were dominated by Proteobacteria, Bacteroidetes and Firmicutes. Although the fed CoTS stomach bacteria were dominated by Tenericutes, it is worth noting that the male gonad microbiota of CoTS was also found to be dominated by Tenericutes in a previous study, despite differences in tissue [27]. The study identified *Endozoicomonas*, *Mycoplasma*, *Pseudomonas* and *Acinetobacter* as the predominant genera. This differs slightly from earlier studies, which reported that the most dominant bacterial types in CoTS were *Endozoicomonas* and *Flavobacteriaceae* [27,30]. The variation could be attributed to differences in growth stage, diets, populations and rearing conditions [47,48,49,50]. Previous research on grass carp has indicated that the composition of gut microbiota changes in response to growth stage, diet and population [47,48,50].

### 4.2. Changes in the Key Bacteria in CoTS Stomach Under Starvation

The bacterial composition in starved CoTS differed from that of fed CoTS, indicating that starvation affects the bacterial population residing in the CoTS stomach. Additionally, the relative abundance of various bacterial taxa changed during the period of starvation. The study found that starvation led to a significant increase in Proteobacteria (including *Endozoicomonas* and *Acinetobacter*), and a sharp decrease in Tenericutes, including *Mycoplasma*. These findings are consistent with the observed changes in the intestinal flora of mice facing starvation [42]. Proteobacteria is considered the most unstable phylum among the four main phyla (Firmicutes, Bacteroidetes, Proteobacteria and Actinobacteria) in the gut microbiota [51]. It responds sensitively to environmental factors and is known as the front-line responder in the gut microbiota [52]. For example, Proteobacteria was more abundant in starved zebrafish [53] and Nile tilapia [42] compared to the nourished state. Proteobacteria blooms are often linked to energy disequilibrium and an unstable gut microbial community structure [52], although this is not always the case. For instance, in a healthy steady state, the relative abundance of Proteobacteria in the human gut can increase to 45% in just 15 months without any clinical signs in the host [54]. Therefore, it is possible that changes could provide the CoTS with a chance to adapt to the new environment by reshaping the stomach microbiota. Previous research has suggested that heterotrophic Proteobacteria have evolved various strategies to undergo the nutrient deprivation and other stresses commonly encountered in natural environments [55].

Chlamydiae are ubiquitously found in various environments, including soli, rhizosphere, sediments, freshwater, wastewater and seawater [56]. They are also present in diverse marine host-associated microbiota, such as molluscs (oyster and mussel), cnidarians (coral and hydra), fish, tunicates, porifera, and Echinodermata [56,57,58]. The study found a significant increase in the relative abundance of Chlamydiae in starved CoTS compared to the fed CoTS, consistent with previous research that also observed a significant increase in the relative abundance of Chlamydiae in the sea urchin intestine following nanoplastic treatment [59]. Chlamydiae were previously recognized as major human pathogens due to their ability to cause the downregulation of immune gene expression through persistent intestinal infection [56]. However, their function and role in other animals remain unclear. The study suggests that Chlamydiae may have a different role in CoTS, as immune-related genes were upregulated in the starved CoTS according to transcriptome results [19].

In contrast, the relative abundance of Tenericutes decreased sharply in starved CoTS. This may be explained by the chronic food deprivation. Tenericutes are a group of endosymbiotic and/or parasitic bacteria without a cell wall. They are often detected in the guts of metazoans [60] and are also the dominant group of cnidarian and seaweed microbiota, including those of gorgonians [61], stony corals [62], jellyfish [63], *Halimeda opuntia* and *Sargassum hemiphyllum* [64]. Furthermore, it has been reported that the dominance of *Mycoplasma* (phylum Tenericutes) members, observed in the digestive systems of nudibranchs, mollusks and other invertebrates, has been suggested to be possibly playing a beneficial role in aiding host digestion [65,66,67,68,69]. In this study, the dominant group in the fed CoTS was the *Mycoplasma* of phylum Tenericutes. This group decreased significantly after starvation, indicating that Tenericutes most likely played a role in digesting food in the fed CoTS stomach. Additionally, the characteristics of the CoTS feeding on stony corals may have further established the dominant role of Tenericutes in the evolutionary history of CoTS stomach flora, as the predator microbiota are closely related to prey microbiota. However, further study is required to determine its specific function and mechanism of action, including whether it can be used as an indicator species to reflect CoTS that are subjected to starvation stress.

At the genus level, starvation resulted in a significant increase in the relative abundance of the genus *Endozoicomonas* (the order Oceanospirillales of the class Gammaproteobacteria), which was also the predominant genus and biomarker in starved CoTS. *Endozoicomonas* has been reported to form associations with a wide range of marine organisms, including cnidarians, poriferans, molluscs, annelids, tunicates, echinoderm and fish [27,70,71,72,73,74,75,76]. The roles of *Endozoicomonas* can be classified into three groups: obtaining and providing nutrients, structuring the host microbiome, and contributing to the health or illness of the host [77]. Therefore, increased *Endozoicomonas* in the starved CoTS may help the host acquire energy and promote nutrient synthesis to cope with food shortages. Several reports suggest that *Endozoicomonas* plays a crucial role in nutrient acquisition, including nitrogen and carbon recycling, or methane and sulfur cycling, and the synthesis of amino acids and other essential molecules [72,73,78,79,80,81]. Thus, it is speculated that the increased *Endozoicomonas* in CoTS under the starvation stress could lead to biosynthesis by organisms through multiple pathways to compensate for the suspension of food supply. Additionally, the result of STAMP and LEfSe analysis showed a significant increase in the presence of the genera *Acinetobacter*, *Halodesulfovibrio*, and *Ralstonia* in the starved group. *Acinetobacter* is believed to be responsible for the breakdown of saponins in the gut, enabling the *Camellia weevil* to survive as a pest in plant fruits and resist higher concentrations of the defensive chemicals [82]. The study suggests that the increase in *Acinetobacter* may also aid the CoTS in enduring harsh environments, such as starvation. *Halodesulfovibrio* is a class of sulfate-reducing bacteria that can use a wide range of electron donors. It contains multiple encoding genes that enable it to obtain organics, which may aid its survival in nutrient-deficient conditions [83,84]. *Ralstonia* is a genus that serves various functions in marine life. It has been identified as a gut microbiota biomarker in the deteriorative process of sea cucumbers [85]. Additionally, it is the dominant genus found in sea urchin gonads [86] and is a core bacterial population in the bundles, planula larvae and parental colonies of the coral *Mussismilia hispida* [87]. Furthermore, it has been reported that *Ralstonia* may have a nitrogen-fixing effect through symbiosis with the host [88]. These findings suggest that the significant increase in Ralstonia observed in the current study may play an important role in CoTS’ response to starvation stress. *Mycoplasma*, which has also been reported to symbiotically aid digestion in the digestive systems of other invertebrates [66], was rapidly reduced in starved CoTS. On one hand, this reduction may be explained by CoTS’ decreased need for bacteria with digestive functions due to food deprivation. On the other hand, food deprivation alone cannot support the dominance of Mycoplasma due to nutrient deficiencies, as the growth of Mycoplasma is dependent on the high abundance of required substrates, such as amino acids, nucleobases, and fatty acids in the host’s digestive tract [89].

### 4.3. Interaction Relationships Between Bacterial Communities by Co-Occurrence Network in the CoTS Stomach

Microbial species’ interactions determine the stability and function of microbial communities, which are closely related to host health [90,91,92]. Network analysis has been widely used to decipher the co-occurrence patterns of microbial communities and their responses to external disturbances [93,94,95]. Previous research has demonstrated that disturbances can alter the complexity and stability of gut microbial networks in aquatic organisms [94,96,97]. Our current study found similar results, with clear shifts in microbial interaction network patterns between the fed and starved groups. Specifically, the fed group exhibited higher network connectivity (i.e., network degree) than the starved group. In the fed group, there are approximately the same number of positive and negative correlations. However, in the starved group, there are significantly more positive correlations than negative correlations. The decrease in negative edges in the network after starvation suggests a reduction in competition between bacteria. This may not be conducive to the network stability as competitive interactions between microbes can have a positive effect on microbiome stability [98,99,100]. In addition, some nodes may not have any direct influence or interaction with one another due to a lack of network connections [101]. This is in stark contrast to the fed group, where all nodes are connected to one another. Furthermore, the difference in node sizes was more noticeable in the starved group than in the fed group, indicating a shift in the microbial composition within the starvation group [90,102]. All of these findings suggest that the microbial communities in the fed group exhibited a more balanced distribution, stability and complexity compared to those in the starved group.

However, it is worth noting that while the network of the starved CoTS was less stable than that of the fed CoTS, it was more modular. Modularity is ubiquitously observed in complex systems in nature, from *Caenorhabditis elegans* to humans [103]. This may be due to the fact that modular architecture reduces metabolic costs by reducing the average length and number of connections, which are the network’s wiring cost [104]. Modularity has the potential to enhance fitness in information processing systems, enabling robust network dynamics. It also allows for the reconfiguration of connections between nodes without compromising information processing function [104,105]. Additionally, modular networks are capable of solving tasks more quickly and accurately, and evolving faster than non-modular networks [106]. The characteristics of network modularity enable systems to evolve rapidly under new selection pressures [106,107]. Therefore, the increased modularity of the stomach microbial network in starved CoTS may be the result of the selective adaptation of the CoTS facing new stress such as starvation. This change may help CoTS maintain their basal metabolic capacity under adverse environmental conditions.

## 5. Conclusions

In this study, 16S rRNA gene high throughput sequencing was used to comprehensively explore the changes in stomach bacterial communities of the CoTS in response to starvation stress. Our result revealed that starvation stress caused a significant change in the stomach bacterial community structure of CoTS, with the stomach flora shifting from digestive bacteria to a community dominated by potentially beneficial bacteria. In addition, starvation reduced the network stability of the stomach bacterial community in CoTS, but increased its modularity, which may be a novel strategy for coping with starvation stress. These findings provide a new way to explore the conservation mechanism of CoTS populations at the end of an outbreak. However, further research is needed to understand the functional mechanism of the differential responders in the CoTS stomach when facing starvation.

## Figures and Tables

**Table 1:** Richness and diversity indices of the 16S rRNA gene from the fed group (n = 2) and the starved group (n = 6) (expressed as the mean ± standard deviation [SD]).

	Fed	Starved	*p*
Richness	189.50 ± 24.75	511.33 ± 226.85	0.071
Shannon_index	1.25 ± 1.25	3.46 ± 1.06	0.071
Pielou	0.24 ± 0.23	0.56 ± 0.18	0.143
Gini_simpson_index	0.40 ± 0.42	0.83 ± 0.15	0.143

**Table 2:** Significant difference tests of the overall stomach bacterial community structure between fed and starved CoTS using three statistical approaches.

Group	Permanova		Anosim		MRPP	
	F	*p*	r	*p*	δ	*p*
Fed vs. starved	0.382	0.042	0.865	0.038	0.793	0.046

**Table 3:** General topological properties of co-occurrence network analysis.

Network Metrics	Fed	Starved
Nodes	50	64
Edges	1275	308
Positive edge	675 (52.94%)	265 (86.04%)
Negative edge	600 (47.06%)	43 (13.96%)
Average degree	51	9.625
Avg. weighted degree	4	7.015
Network diameter	1	8
Graph density	1.041	0.153
Modularity	0	0.559
Connected components	1	7
Avg. clustering coefficient	0.989	0.647
Avg. path length	1	2.878

## Data Availability

The datasets generated and analyzed during the current study are available from the corresponding authors on reasonable request. Datasets supporting 16S rRNA gene sequencing data of this article have been deposited in the NCBI SRA under accession code SUB14330292.

## Supplementary Materials

The following supporting information can be downloaded at: https://www.mdpi.com/article/10.3390/biology14081102/s1, Figure S1: The composition of the bacterial community in the stomach of each CoTS sample at the phylum and genus level; Table S1: General parameters characteristic of co-occurrence network nodes in fed CoTS; Table S2: General parameters characteristic of co-occurrence network nodes in starved CoTS.
