# Supplementary material for: Starvation Influences the Microbiota in the Stomach of the Corallivorous Crown-of-Thorns Starfish
Source: Biology (Basel). 2025 Aug 21;14(8):1102. doi: 10.3390/biology14081102 (PMC12383813; doi:10.3390/biology14081102)
Supplement: Supplementary file 1 [file biology-14-01102-s001.zip › Supplement materials.pdf]

Starvation Influences the Microbiota in the Stomach of the Corallivorous Crown-of-Thorns Starfish

Ying Zhang [1,†], Fuxiang Lai [1,3,†], Litong Yang [1], LiLing Dai [1,3], Nan Su [1,4], Jianxing Hu [1], Huizhen Chen [1], Qian Gao [1], Fanyu Zheng [1] and Chang Chen [1,2,*]

1 CAS Key Laboratory of Tropical Marine Bio-Resources and Ecology, Guangdong Provincial Key Laboratory of Applied Marine Biology, South China Sea Institute of Oceanology, Chinese Academy of Sciences, Guangzhou 510301, China
2 Xisha Marine Environment National Observation and Research Station, South China Sea Institute of Oceanology, Chinese Academy of Sciences, Sansha 573199, China
3 College of Marine Sciences, South China Agricultural University, Guangzhou 510640, China
4 Institute of Hydrobiology, Jinan University, Guangzhou 510632, China
* Correspondence: chen.chang@scsio.ac.cn; Tel.: +86-186-2000-6618
† These authors contributed equally to this work.

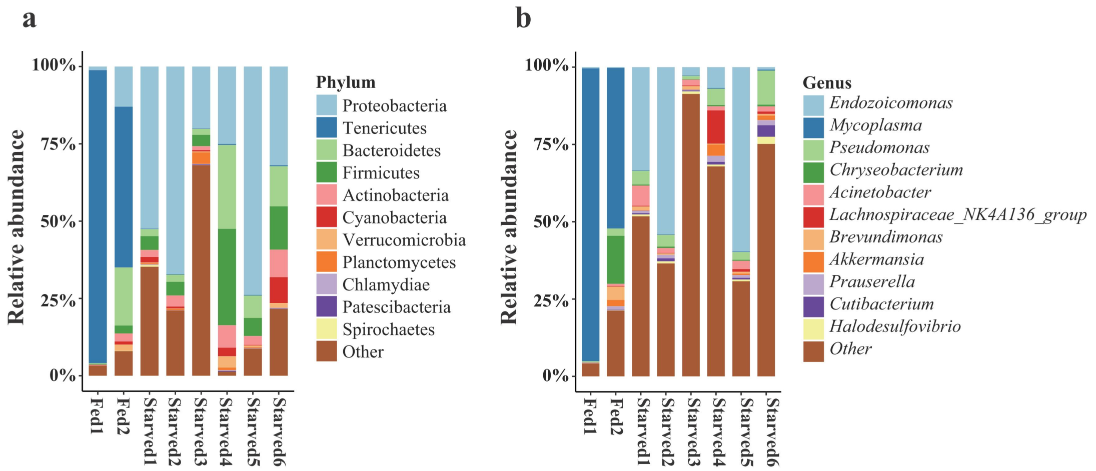

Figure S1. The composition of the bacterial community in the stomach of each CoTS sample at the phylum and genus level.
